# The Dynamic Interactions of a Multitargeting Domain in Ameloblastin Protein with Amelogenin and Membrane

**DOI:** 10.3390/ijms24043484

**Published:** 2023-02-09

**Authors:** Natalie C. Kegulian, Ralf Langen, Janet Moradian-Oldak

**Affiliations:** 1 Center for Craniofacial Molecular Biology, Department of Biomedical Sciences, Herman Ostrow School of Dentistry, University of Southern California, Los Angeles, CA 90033, USA; 2 Department of Neuroscience and Physiology, Department of Biochemistry and Molecular Medicine, Zilkha Neurogenetic Institute, Keck School of Medicine, University of Southern California, Los Angeles, CA 90033, USA

**Keywords:** ameloblastin, membrane binding, amelogenin, biomineralization, electron paramagnetic resonance (EPR), fluorescence spectroscopy

## Abstract

The enamel matrix protein Ameloblastin (Ambn) has critical physiological functions, including regulation of mineral formation, cell differentiation, and cell–matrix adhesion. We investigated localized structural changes in Ambn during its interactions with its targets. We performed biophysical assays and used liposomes as a cell membrane model. The xAB2N and AB2 peptides were rationally designed to encompass regions of Ambn that contained self-assembly and helix-containing membrane-binding motifs. Electron paramagnetic resonance (EPR) on spin-labeled peptides showed localized structural gains in the presence of liposomes, amelogenin (Amel), and Ambn. Vesicle clearance and leakage assays indicated that peptide–membrane interactions were independent from peptide self-association. Tryptophan fluorescence and EPR showed competition between Ambn–Amel and Ambn–membrane interactions. We demonstrate localized structural changes in Ambn upon interaction with different targets via a multitargeting domain, spanning residues 57 to 90 of mouse Ambn. Structural changes of Ambn following its interaction with different targets have relevant implications for the multifunctionality of Ambn in enamel formation.

## 1. Introduction

The process of biomineralization to form enamel, dentin, bone, and other hard biomaterials depends upon the coordinated action of extracellular matrix proteins [1,2]. Enamel matrix protein (EMP) interactions with each other and with ameloblasts, the epithelial cells that secrete them, are critical for proper tooth enamel formation [3,4,5,6,7]. Mutations in the genes encoding the major structural proteins in enamel—namely amelogenin (Amel), ameloblastin (Ambn), enamelin (Enam), and amelotin (Amtn)—are associated with amelogenesis imperfecta (AI) [8,9,10,11], a hereditary disorder characterized by hypoplastic (thin) and/or hypomineralized (weak) enamel [12].

Ambn is an intrinsically disordered protein [13] belonging to the secretory calcium-binding phosphoprotein (SCPP) family [14]. After Amel, it is the second-most abundant component of structural EMP content [15]. During the secretory stage of amelogenesis, ameloblasts secrete Amel, Ambn, and Enam into the extracellular matrix space, where these EMPs guide the formation of enamel rods from the crystallization of hydroxyapatite [16]. EMPs are subsequently digested by proteases and removed, leaving behind hard mature enamel with >95% mineral content [17,18]. A growing body of evidence suggests that Amel serves as a template for the correct mineral phase, crystal morphology, and organization of hydroxyapatite in enamel rods [19,20,21]. The specific role of Ambn has not been as widely studied and appears to be multipronged, comprising adhesion [3], signaling [22], mineral nucleation [23], and control of prismatic structure, possibly via its interaction with Amel [6,24].

Recent in vitro and cell culture studies indicated that an evolutionarily conserved amino acid sequence within the N-terminal region of exon 5 of Ambn interacts with the cell membrane [7,25]. This sequence forms an amphipathic helix (AH) in the presence of phospholipid bilayer membrane vesicles in vitro [25] and binds to ameloblast-like cell plasma membranes [7]. A comprehensive study of Ambn’s secondary structure across animal species each carrying prismatic, nonprismatic, or no enamel showed that prismatic enamel formation strongly correlates to this AH-forming sequence and its ability to interact with membranes [26]. Remarkably, the N-terminal region of Ambn exon 5 also includes an evolutionarily conserved Y/F-x-x-Y/L/F-x-Y/F motif that mediates self-assembly in Amel, Ambn, and other intrinsically disordered proteins [27] and could conceivably drive Amel–Ambn co-assembly. Collectively, studies uncovering Ambn–membrane [7,25,26] and Amel–Ambn [28,29,30] interactions support the notion that the N-terminal region of Ambn exon 5 associates both with membranes and with Amel in addition to being involved in Ambn self-assembly.

The present study explores Ambn–Amel and Ambn–liposome interactions and the dynamics between them, providing insight into the simultaneous occurrence of these interactions and the physiological function of Ambn. We used liposomes as a cell membrane model and, for specific mimicry of the ameloblast membrane, prepared them using a composition resembling that of the membrane domain involved in epithelial cell-extracellular matrix adhesion [31]. To elucidate the structures of the membrane-binding and assembly-mediating regions of Ambn, we used electron paramagnetic resonance (EPR) with site-directed spin-labeling (SDSL) combined with circular dichroism (CD), fluorescence spectroscopy, membrane leakage assays, and optical absorbance spectroscopy. Our experiments utilized a series of synthetic peptides derived from the regions of Ambn encoded by exons 3, 4, and 5. Splitting the Ambn regions of interest into these peptides prevented aggregation and allowed us to focus on the local structural changes in these residues upon interactions. We detected conformational changes at specific Ambn sites during interactions by using a combination of spectroscopic methods. These techniques included EPR with SDSL, an excellent technique for filling knowledge gaps regarding local structures within proteins of varying levels of structural order [32,33,34,35,36]. Techniques such as CD only detect global conformational changes. The EPR technique is advantageous because it does not require high concentrations [37], immobilization [38], or freezing of samples [39]. EPR has been used extensively on other proteins to interrogate protein–membrane [40,41], protein–protein [42,43], and protein–co-solute [44] interactions at a site-specific resolution. While overall Ambn structure has been investigated previously [25,45,46], this is the first time that localized structure at an Ambn domain has been identified in the presence of multiple targets.

## 2. Results

### 2.1. Characterization of Spin-Labeled Ambn Peptides

We employed a variety of complementary biophysical techniques (EPR, CD, fluorescence, optical absorbance) to identify any conformational changes occurring in Ambn-derived peptides AB2 and xAB2N (Table S1) during their interactions with different targets. First, we evaluated the overall secondary structures of the spin-labeled peptides in the presence and absence of epithelial cell membrane composition-mimicking liposomes, to be hereby referred to as large unilamellar vesicles (LUVs), using CD. AB2—spin-labeled at positions 5, 10, 12, 19, 24, and 35 (to be referred to as AB2-5R1, AB2-10R1, and so forth)—was evaluated. These CD measurements (Figure S1A–F) were taken to ascertain that the secondary structures of these spin-labeled AB2 cysteine mutants in the presence and absence of LUVs did not deviate from the structures of wild-type AB2 [25]. The coil–helix transition previously measured in detail via CD on AB2 and full-length Ambn [25] was observed in each spin-labeled mutant. The small peak of each spectrum from peptide alone at 208 nm and the accompanying shoulder at 222 nm became more pronounced minima upon the addition of LUVs (Figure S1, blue traces), a profile typical of increased α-helical content. Spectra overall appeared redshifted in the presence of LUVs compared to spectra in their absence (Figure S1, black traces), supporting a coil-helix transition. We measured the CD spectra of xAB2N, as well as of its spin-labeled variants xAB2N-7R1, xAB2N-15R1, and xAB2N-18R1. All four peptides showed an increase in α-helical content in the presence of LUVs as signified by the more pronounced downward peaks at 208 and 222 nm (Figure S1G–J), which matched the changes previously observed in AB2 upon interaction with membranes.

### 2.2. EPR Revealed That Ambn Peptides Gain Secondary Structure upon Interaction with Liposomes

**Figure 1:**
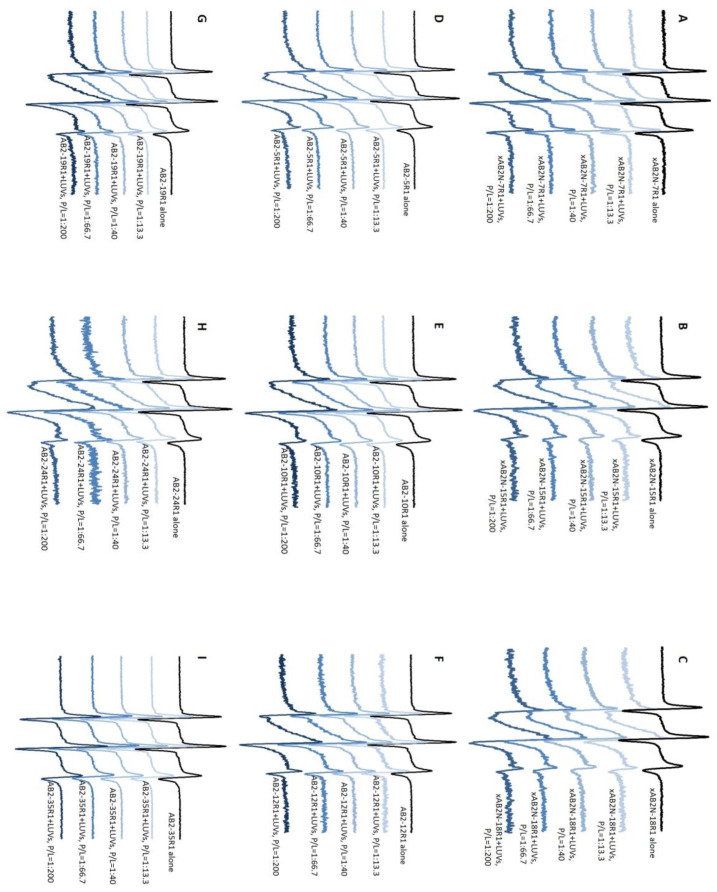
Representative EPR spectra of 15 μM peptide with LUVs at indicated peptide-to-phospholipid molar ratios. All EPR spectra, in this and subsequent figures, are renormalized to the same amplitude. xAB2N spin-labeled at positions (**A**) 7, (**B**) 15, and (**C**) 18 and AB2 spin-labeled at positions (**D**) 5, (**E**) 10, (**F**) 12, (**G**) 19, (**H**) 24, and (**I**) 35.

The EPR spectra of spin-labeled AB2 and xAB2N showed localized structural changes in the N-terminal region of AB2 and throughout xAB2N in the presence of membranes. Eight of the nine peptides examined in EPR—namely xAB2N-7R1, xAB2N-15R1, xAB2N-18R1, AB2-5R1, AB2-10R1, AB2-12R1, AB2-19R1, and AB2-24R1—showed a dose-dependent broadening of spectral lines upon addition of LUVs (Figure 1A–H), which indicates an increase in secondary structure. In contrast, AB2-35R1, which—like all other labeled peptides—formed an α-helix detected by CD in the presence of LUVs (Figure S1F), showed no spectral broadening in EPR, even at the highest lipid concentration (Figure 1I).

**Figure 2:**
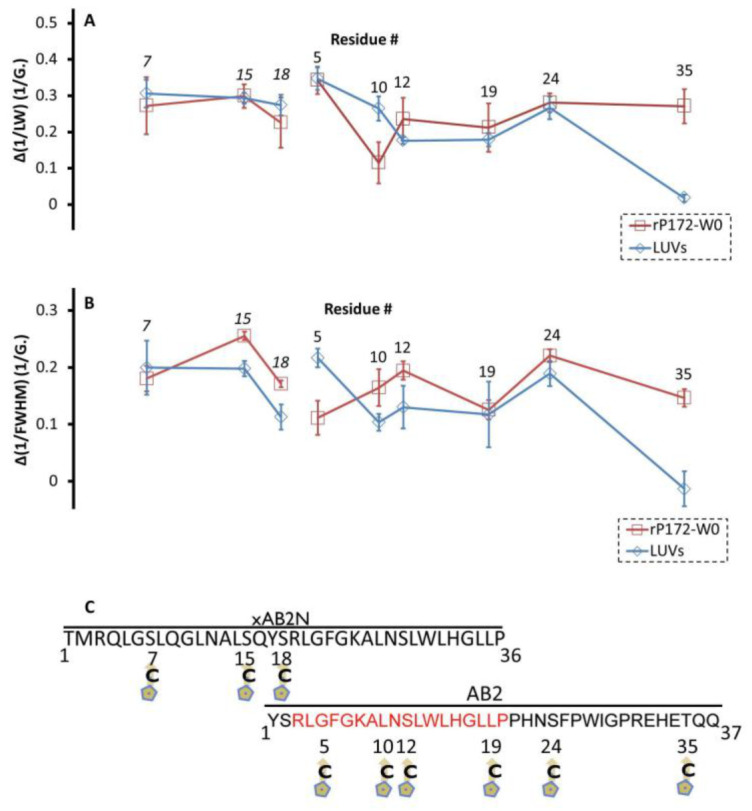
Changes in (**A**) inverse central linewidth (1/LW) and (**B**) inverse full width at half maximum (1/FWHM) in EPR spectra of 15 μM spin-labeled AB2 or xAB2N between spectra without and with 3 mM LUVs or 75 μM rP172-W0. Error bars represent standard errors of the mean (*n* = 3). Peptides with amino acids that were singly mutated to cysteine and spin-labeled are shown underneath in (**C**), with each labeled residue aligned with its position on the graph. Residue numbers correspond to xAB2N and AB2 peptide sequences; residues 1–36 of xAB2N corresponds to residues 51–86 of mouse Ambn, and residues 1–37 of AB2 correspond to residues 67–103 of mouse Ambn. Residues 3–20 of AB2, designating the amino acids previously predicted to form an AH upon interaction with membrane (25), are in red font.

We analyzed this spectral broadening for each EPR spectrum by measuring its central linewidth (LW), which is the distance in Gauss (G.) between the centers of the highest and lowest peaks, and by plotting the inverse central linewidths (1/LW) as measures of mobility (Figure 2A). The differences between inverse central LWs of spectra generated from spin-labeled peptides with and without LUVs were consistently between 0.17 and 0.35 G.^−1^ for AB2 labeled at positions 5, 10, 12, 19, and 24 and xAB2N labeled at positions 7, 15, and 18 (Figure 2A, blue trace). In contrast, the difference between inverse central LWs of AB2-35R1 with and without LUVs was 0. A similar pattern was observed using the full width at half maximum (FWHM) method (Figure 2B). The sequences of the peptides are shown in Figure 2C, with the labeled residues aligned with the charts in Figure 2A,B.

### 2.3. EPR Revealed That Ambn Peptides Gain Tertiary/Quaternary Contacts upon Interaction with Full-Length Amel

**Figure 3:**
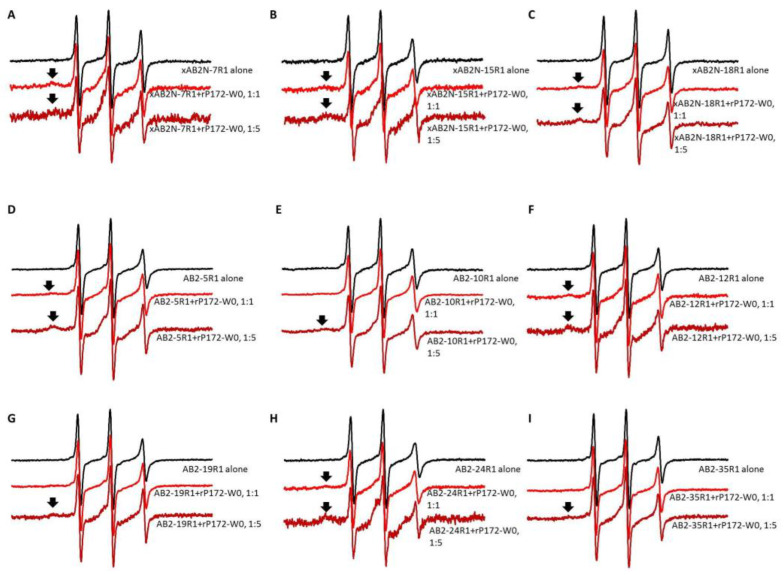
Representative EPR spectra of 15 μM peptide with rP172-W0 at indicated peptide-to-protein molar ratios. Arrows denote immobile spectral components. xAB2N spin-labeled at positions (**A**) 7, (**B**) 15, and (**C**) 18 and AB2 spin-labeled at positions (**D**) 5, (**E**) 10, (**F**) 12, (**G**) 19, (**H**) 24, and (**I**) 35.

The Amel–Ambn co-assembly domain (YSRLGF) identified previously [28] appears at the N-terminal end of AB2 and in the middle of xAB2N. Thus, we investigated whether a similar alteration in local structure as that observed in response to LUVs occurred in Ambn-derived peptides due to interaction with Amel. Upon titration of spin-labeled xAB2N and AB2 with tryptophan-free recombinant porcine Amel (rP172-W0) (Table S1), we observed spectral broadening at all labeled sites (Figure 3A–I), even AB2-35R1 (Figure 3I). This finding signifies a change in local structure throughout xAB2N and AB2 in response to Amel. In addition, most spectra measured in the presence of rP172-W0 showed an immobile spectral component (bump designated by black arrows in Figure 3). The strong immobilization associated with this peak is characteristic of the residue in each case being a tertiary or quaternary contact site. The differences between inverse central LWs of spectra generated with and without Amel were consistently between 0.16 and 0.35 G.^−1^ for all residues (Figure 2A, red trace), similar to the changes observed between spectra in the absence and in the presence of LUVs. The differences between inverse FWHMs of EPR absorbance spectra generated with and without Amel were also similar to those generated with and without LUVs (Figure 2B). However, the immobilization of xAB2N and AB2 is much greater in the presence of Amel than of LUVs. This is based on the spectra measured after LUV addition, which lack the immobile spectral component, or bump, present in spectra measured after Amel addition. Titration of spin-labeled xAB2N or AB2 with bovine serum albumin (BSA), used as a negative control, did not result in a broadening of peaks in EPR (Figure S2A–I), indicating that the change in local conformations of xAB2N and AB2 in the presence of Amel was specifically caused by Amel and not by nonspecific protein–peptide interactions.

### 2.4. EPR Revealed That Ambn Peptides Gain Structural Order upon Interaction with Full-Length Ambn

**Figure 4:**
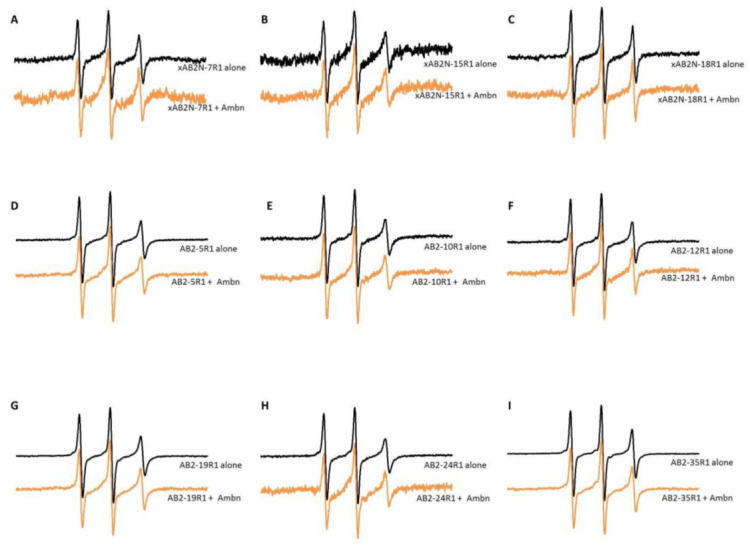
Representative EPR spectra of 15 μM peptide with and without 0.3 mg/mL unlabeled full-length Ambn. xAB2N spin-labeled at positions (**A**) 7, (**B**) 15, and (**C**) 18 and 15 µM AB2 spin-labeled at positions (**D**) 5, (**E**) 10, (**F**) 12, (**G**) 19, (**H**) 24, and (**I**) 35.

The YSRLGF sequence in AB2 and xAB2N peptides that mediates Amel–Ambn co-assembly is also the Y/F-x-x-Y/L/F-x-Y/F self-assembly domain previously identified in Ambn [27,28]. To assess whether a similar alteration in local structure as that observed in response to LUVs and Amel occurs due to self-interaction, we added full-length Ambn to spin-labeled xAB2N and AB2. We observed a broadening of peaks in all labeled residues of both AB2 and xAB2N (Figure 4A–I) that was especially noticeable for xAB2N-7R1, which is close to the Ambn self-assembly motif. Therefore, EPR indicated that the addition of full-length Ambn induces local structural changes throughout the residues in Ambn encoded by exon 5 and more drastic changes in residues N-terminal to those residues. To evaluate the structural effect of self-interaction under conditions closer to the high Ambn concentrations in vivo, we titrated spin-labeled xAB2N and AB2 with unlabeled AB2 but found only minor spectral change, most noticeably for xAB2N-7R1 (Figure S3A–I).

### 2.5. Experimental Scenarios to Examine Simultaneous Ambn Peptide Interactions with Its Targets

**Figure 5:**
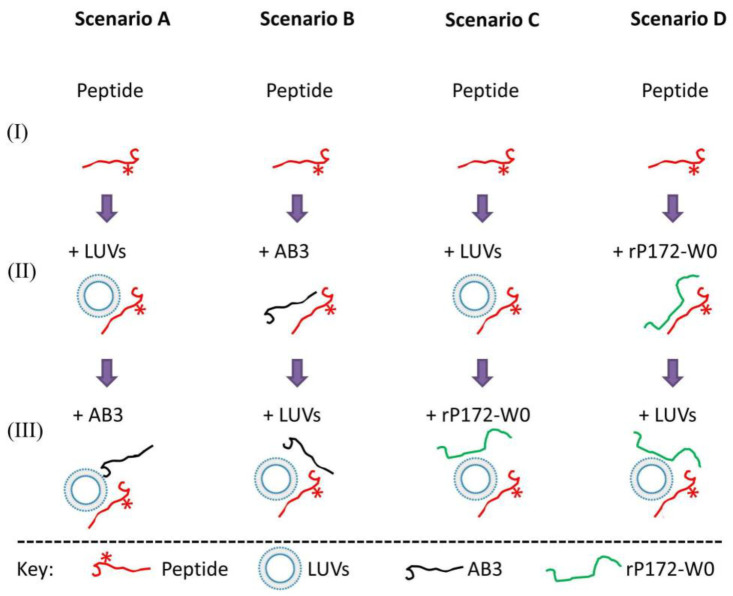
Schematic representation for three-component systems used in tryptophan fluorescence measurements in and EPR experiments in. First, AB2, AB2N, xAB2N, or AB2N-W14Y was measured in the absence of another component (I). In Scenario A, LUVs were subsequently added, and fluorescence spectra were measured again (II). Then, AB3, which does not contain tryptophan residues, was added to the mixture, and a third measurement was made (III). In Scenario B, AB3 was added to the tryptophan-containing peptide first (II), followed by LUVs (III). Scenarios C and D are the counterparts to Scenarios A and B, respectively, with rP172-W0, or non-tryptophan-containing Amel, added in place of AB3.

To examine the dynamics of interactions between three different components—Ambn peptides, LUVs, and either additional Ambn peptide or Amel—we used intrinsic tryptophan fluorescence spectroscopy. We measured changes in the spectra of AB2, AB2-W14Y, AB2N, and xAB2N peptides in the presence or absence of LUVs, of tryptophan-free AB2 peptide (AB3), and/or of rP172-W0. We provide a scheme (Figure 5) describing our experiments combining tryptophan-containing peptide, LUVs, and rP172-W0 or AB3. Intrinsic tryptophan fluorescence was measured for AB2, AB2N, xAB2N, or AB2-W14Y in buffer alone (I). For Scenarios A and C, LUVs were subsequently added, and the fluorescence was measured again (II). For Scenario B, AB3 was added instead at step (II); for Scenario D, rP172-W0 was added. Finally, the third component was added, and a third measurement was taken (III). This third component was AB3 for Scenario A, LUVs for Scenarios B and D, and rP172-W0 for Scenario C. These three-way systems tested whether AB2 and related peptides could interact with both membranes and proteins simultaneously or competitively.

### 2.6. Fluorescence Spectroscopy Showed Concomitant Ambn Self-Association and Ambn–Membrane Interaction

**Figure 6:**
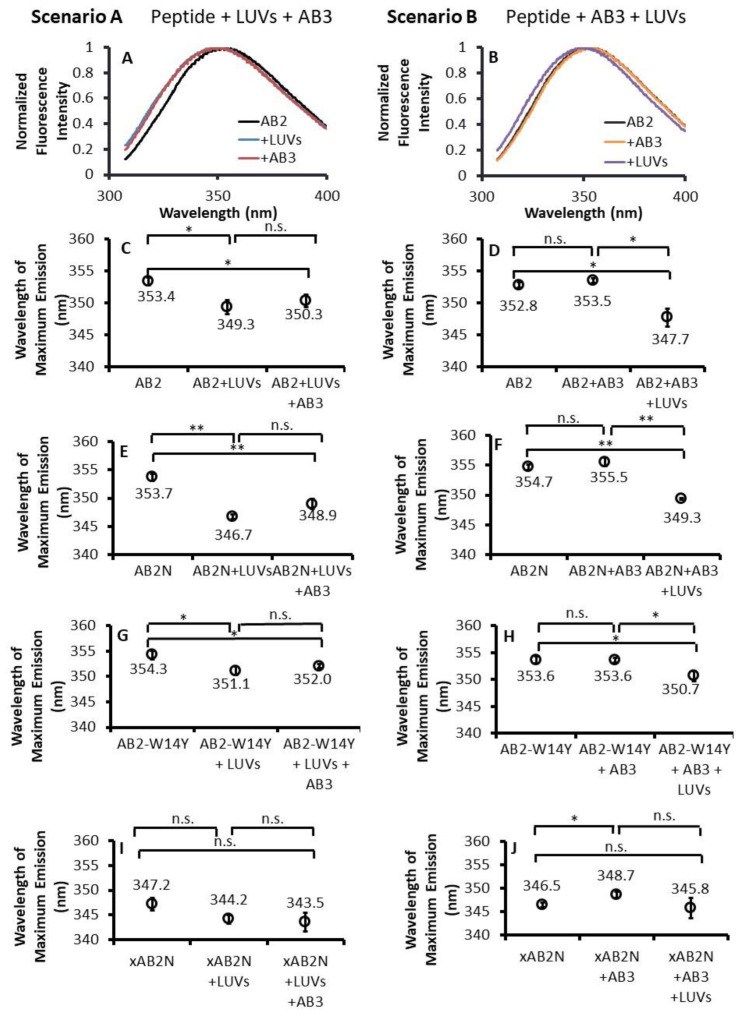
Intrinsic tryptophan fluorescence on Scenarios A and B depicted in. Representative spectra (**A**,**B**) and wavelengths of maximum emission (**C**,**D**) from 8 μM AB2 with 240 μM LUVs, then with 40 μM AB3 subsequently added (**A**,**C**), and 8 μM AB2 with 40 μM AB3, then with 240 μM LUVs subsequently added (**B**,**D**). Wavelengths of maximum emission from (**E**) 8 μM AB2N with 240 μM LUVs, then with 40 μM AB3 subsequently added; (**F**) 8 μM AB2N with 40 μM AB3, then with 240 μM LUVs subsequently added; (**G**) 8 μM AB2-W14Y with 240 μM LUVs, then with 40 μM AB3 subsequently added; (**H**) 8 μM AB2-W14Y with 40 μM AB3, then with 240 μM LUVs subsequently added; (**I**) 8 μM xAB2N with 240 μM LUVs, then with 40 μM AB3 subsequently added; and (**J**) 8 μM xAB2N with 40 μM AB3, then with 240 μM LUVs subsequently added. Representative spectra for conditions in (**E**–**J**) shown in Figure S4. One-tailed Student’s *t*-test; *n* = 3; ** *p* < 0.01; * *p* < 0.05; n.s., not significant. Error bars represent standard errors of the mean.

To compare AB2–membrane and AB2–AB2 interactions, we conducted fluorescence spectroscopy experiments exploiting the intrinsic fluorescence of the two tryptophan residues in Ambn exon 5 (underlined in Table S1). When LUVs were added at a 1:30 peptide-to-lipid molar ratio, there was a decrease in the wavelength at which AB2 tryptophan fluorescence emission peaked (Figure 6A,C). This blueshift is in step with previous AB2–LUV interaction data [25] and indicates a movement of tryptophan residues into a more hydrophobic microenvironment [47]. To determine whether adding more AB2 affects the extent of this interaction by increasing peptide–peptide interactions, we added the tryptophan-free peptide AB3 at a 1:30:5 AB2-to-lipid-to-AB3 molar ratio. We detected a trend toward a redshift in emission peak (Figure 6A,C), which indicates a more hydrophilic microenvironment surrounding the tryptophan residues, which agrees with the behavior of tryptophan residues in Amel upon interaction with AB3 [30]. However, unlike the shift that occurred in response to the addition of LUV, this trend toward a redshift upon the addition of AB3 was not statistically significant. We also tested AB2–AB3 interactions by adding AB3 to AB2 in the absence of LUVs at a 1:5 AB2-to-AB3 ratio (Figure 6B,D). We again observed a trend toward a redshift that was not statistically significant. We subsequently added LUVs at the ratio used previously and detected a significant blueshift (Figure 6B,D) similar to the one observed in the absence of AB3. Repeating this experiment using a shorter peptide comprising only the first 20 amino acids of AB2 (AB2N; Figure 6E,F; spectra shown in Figure S4A,B) or full-length AB2 lacking the tryptophan residue in the N-terminal region (AB2-W14Y; Figure 6G,H; spectra shown in Figure S4C,D) yielded similar results. However, there was no trend of a shift in the tryptophan fluorescence peak when AB3 was added to AB2-W14Y, a result that accorded with the placement of the self-assembly domain at the N-terminal end of AB2 [27]. Repeating the experiment using xAB2N showed the same trends as with AB2 and AB2N (Figure 6I,J; spectra shown in Figure S4E,F), but there was no statistical significance in any direction except for a redshift upon addition of AB3 in the absence of LUVs, which was not recapitulated in their presence. This lack of consistent shifts in xAB2N tryptophan fluorescence might indicate either the preformation of xAB2N aggregates or the shielding of this peptide’s lone tryptophan residue by the N-terminal amino acids not present in the other peptides. Since AB2-W14Y is a variant not used in previous studies, its CD was measured in both the absence and the presence of LUVs (Figure S4G) to verify that its conformational behavior matches that established for AB2 [25].

### 2.7. AB2–Membrane Interactions Are Independent of AB2 Self-Association

**Figure 7:**
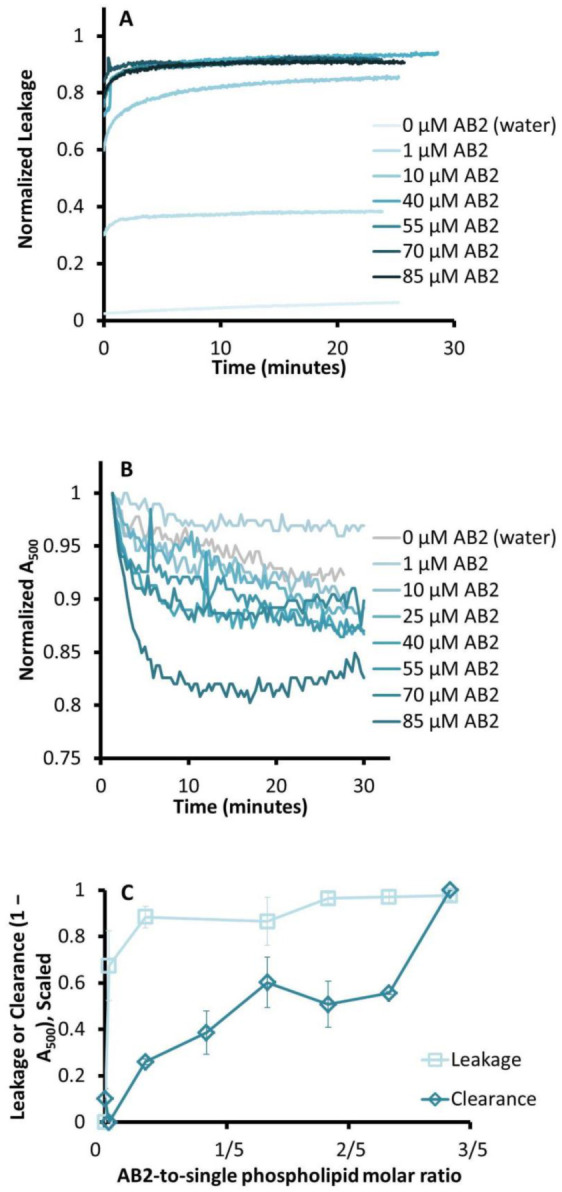
Leakage of 150 μM LUVs (**A**) and static light scattering of 150 μM MLVs (**B**) with indicated concentrations of AB2. (**C**) MLV clearance, defined as light scattering loss (blue), and LUV leakage (light blue) at 15 min upon addition of AB2 to vesicles at the indicated peptide-to-phospholipid molar ratios. Error bars represent standard errors of the mean (*n* = 3).

We next inquired into whether AB2 self-association facilitates AB2–membrane interaction. A protein or peptide binding a membrane is in some cases aided by an oligomerization mechanism stabilizing formation of a protein pore on the membrane [48]. Such a mechanism could entail AB2 oligomer formation, aggregation, or simply proximity of some AB2 monomers to others already bound to the membrane leading to greater ease in binding of more AB2 monomers. We used LUV leakage and multilamellar vesicle (MLV) clearance assays (representative measurements shown in Figure 7A,B) to quantify AB2–membrane interaction at different concentrations of AB2. The relationship between membrane interaction, quantified as either leakage or clearance, and the AB2-to-phospholipid ratio resembled a root function for leakage and a linear function for clearance (Figure 7C) and not an exponential function, as a cooperative mechanism would entail [49]. Specifically, plotting the intensity of leakage versus AB2-to-lipid ratio (Figure 7C, light blue trace) yielded a curve that reached saturation at 10 µM AB2. Leakage, unlike clearance, does not require membrane disintegration and thus can occur and reach saturation at lower concentrations of peptide. Accordingly, plotting the intensity of clearance versus AB2-to-lipid ratio (Figure 7C, blue trace) did not reach saturation within the range of AB2 concentrations tested. The clearance curve also did not point to a threshold AB2 concentration above which clearance increased more markedly than below it, and nor did leakage, which merely reached saturation after which the intensity did not significantly increase. A threshold concentration would arise if direct cooperation among AB2 molecules were required for vesicle clearance or leakage, which would be enabled at a particular concentration of AB2 that optimizes AB2 self-association [48]. Since no such threshold was found, AB2–membrane and by extension Ambn–membrane interactions are not dependent on Ambn self-assembly.

### 2.8. Ambn has Dynamic Interactions with Amel and Membranes near Its Self-Assembly Region

**Figure 8:**
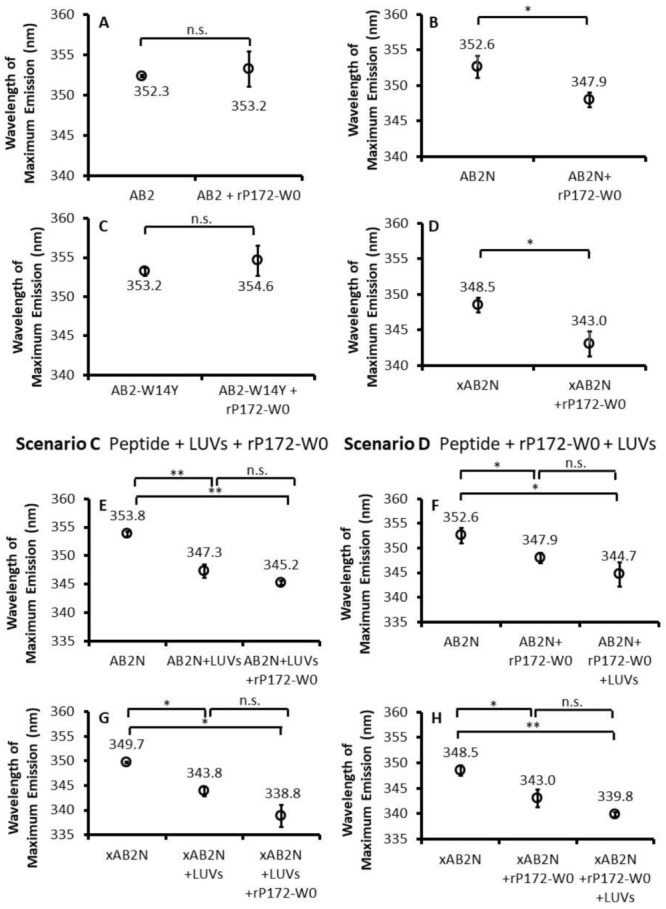
Wavelengths of maximum intrinsic tryptophan fluorescence emission from 8 µM (**A**) AB2, (**B**) AB2N, (**C**) AB2-W14Y, and (**D**) xAB2N before and after addition of 40 µM rP172-W0. Wavelengths of maximum emission from 8 µM AB2N with (**E**) first 240 µM LUVs added in, then 40 µM rP172-W0, as in Scenario C in, and with (**F**) first 40 µM rP172-W0 added in, then 240 µM LUVs, as in Scenario D in. Wavelengths of maximum emission from 8 µM xAB2N with (**G**) first 240 µM LUVs added in, then 40 µM rP172-W0, as in Scenario C in, and with (**H**) first 40 µM rP172-W0 added in, then 240 µM LUVs, as in Scenario D in. Representative spectra for each condition shown in Figure S5. One-tailed Student’s *t*-test; *n* = 3; ** *p* < 0.01; * *p* < 0.05; n.s., not significant. Error bars represent standard errors of the mean.

We have reported that Ambn, AB2, and AB2N peptides interact with epithelial cell-mimicking membranes and with Amel [25,26,28]. The present study confirms the same for xAB2N. We therefore investigated whether Amel and phospholipid membranes could bind Ambn, as represented by AB2 and derivative peptides, simultaneously or one interaction abrogated the other. To this end, we performed both tryptophan fluorescence and EPR experiments using the three-component systems in Scenarios C and D (Figure 5). Adding rP172-W0, which lacks tryptophan (Table S1), to AB2 did not yield a statistically significant shift in the tryptophan fluorescence peak from AB2 (Figure 8A; spectra shown in Figure S5A). We therefore combined AB2N, AB2-W14Y, or xAB2N with rP172-W0 and found a statistically significant blueshift to occur in the peaks generated by AB2N and by xAB2N (Figure 8B,D; spectra shown in Figure S5B,D) but no significant shift in the peak from AB2-W14Y (Figure 8C; spectra shown in Figure S5C). Unlike the redshift in response to addition of AB3 (Figure 6F,J), the blueshifts in AB2N and xAB2N are due to the exposure of tryptophan to a more hydrophobic environment generated by the largely hydrophobic composition of residues in Amel, particularly in its central core region [50]. The fluorescence shifts in AB2N and xAB2N and the lack of florescence shift in AB2-W14Y indicate that AB2 interactions with Amel occur at the N-terminal region of AB2, in agreement with previous co-immunoprecipitation findings [28].

Since AB2N and xAB2N were the only peptides with a significant change in tryptophan fluorescence peak wavelength in the presence of Amel, we selected them for the implementation of Scenarios C and D from Figure 5. Adding LUVs to AB2N (Figure 8E; spectra shown in Figure S5E) or xAB2N (Figure 8G; spectra shown in Figure S5G) combined with rP172-W0, as in Scenario C, led to an increase in the tryptophan blueshift, but it was not statistically significant. Similarly, adding rP172-W0 to AB2N (Figure 8F; spectra shown in Figure S5F) or xAB2N (Figure 8H; spectra shown in Figure S5H) combined with LUVs, as in Scenario D, led to a continuing trend in the tryptophan blueshift, but also without statistical significance, suggesting that Amel competes with LUVs in interacting with Ambn. These results support that AB2N and xAB2N are less available to interact with LUVs while interacting with Amel as well as less available to interact with Amel while interacting with LUVs.

**Figure 9:**
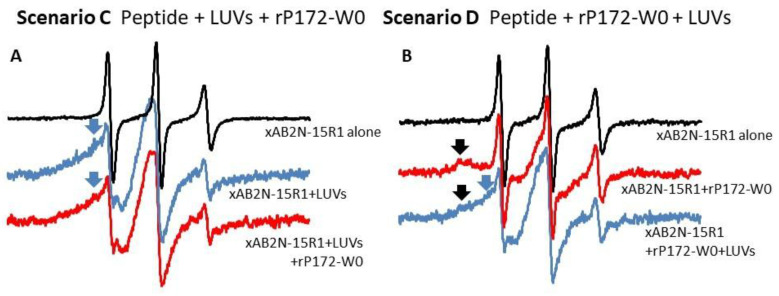
(**A**) EPR spectra of 15 µM xAB2N spin-labeled at position 15 in 10 mM Tris, pH 7.4, 50 mM NaCl, with 600 µM LUVs, followed by 15 µM rP172-W0, added in, as in Scenario C depicted in. (**B**) EPR spectra of 15 µM xAB2N spin-labeled at position 15 in 10 mM Tris, pH 7.4, 50 mM NaCl, with 15 µM rP172-W0, followed by 600 µM LUVs, added in, as in Scenario D depicted in. Black arrows indicate the immobilized spectral component characteristic of protein contacts; blue arrows indicate the intermediately immobilized spectral component characteristic of peptide–LUV interaction.

Comparing the magnitude of a tryptophan blueshift in the presence of both LUVs and Amel to the magnitudes of the shift upon addition of the individual components does not distinguish clearly between their effects. This is partly because the measurement is one-dimensional and unidirectional, with the wavelength of the peak decreasing whether the causative agent is LUVs or Amel. Thus, we performed EPR, whose spectral features contain components such as the immobile spectral components in Figure 3 that distinguish peptide–Amel from peptide–LUV interactions. We chose a spin-labeled site, namely xAB2N-15R1, that had shown strong interactions with both membrane vesicles and Amel and that lies adjacent to the Ambn self-assembly/co-assembly domain, and we subjected this peptide to Scenarios C and D. First, adding LUVs to xAB2N-15R1 at a 1:40 peptide-to-single phospholipid molar ratio resulted in broadening of peaks and a shoulder showing the intermediate immobilization component expected for membrane binding (Figure 9A, blue trace, blue arrow). This spectrum remained largely unchanged when Amel was added at a 1:1 ratio with xAB2N-15R1 (Figure 9A, red trace), indicating that little protein binding occurred in the presence of preexisting membrane interaction. When Amel was added first to this peptide at a 1:1 ratio (Figure 9B, red trace), a much more immobilized spectral component emerged than was expected for protein contacts (black arrow). Addition of LUVs at a 1:40 peptide-to-lipid ratio decreased both this highly immobile component and the residual sharp lines that were caused by peptides that remained unbound after Amel addition (blue trace). Both of these components gave way to the intermediate immobilization that is characteristic of peptide–LUV interaction (blue arrow over blue trace). Thus, the protein contacts appeared to have been broken in order to accommodate the structure of the membrane-bound state, which, under the present conditions, was more favorable. Taken together, these results show Ambn–Amel and Ambn–membrane interactions to be dynamic rather than static and suggest that, when all three are present, competition occurs between Ambn–Amel and Ambn–membrane interactions.

## 3. Discussion

This study seeks to determine localized structural changes in regions of Ambn known to interact with multiple targets and to identify dynamics between interactions of Ambn with Amel and with membranes. We rationally designed peptides AB2 and xAB2N, as well as unlabeled and spin-labeled variants, to encompass residues encoded by exons 3, 4, and 5 of Ambn; used LUVs as a membrane model; and performed a variety of biophysical methods to identify localized changes. EPR revealed localized structural changes in xAB2N and AB2 upon the addition of LUVs in a dose-dependent manner. Previously, predictive software suggested a short and weak helix from residues 3 to 20 of AB2 in the presence of membranes [25], but the start and end points of this helix in the sequence was never tested in vitro. Here, EPR revealed that an immobilized structure forms in the presence of LUVs at least from residues 5 to 24 of AB2 (Figure 1D–H) and not at residue 35 (Figure 1I), a framework that approximately fits the bioinformatics prediction of AH formation from residues 3 to 20 [25]. Structure formation also occurs in residues N-terminal to those of AB2, as shown by EPR data on xAB2N with LUVs (Figure 1A–C). CD spectra showed that this LUV-mediated AB2 and xAB2N structure formation stems at least mainly from α-helical formation (Figure S1). Therefore, the structure formation observed in N-terminal residues of xAB2N by EPR could originate from α-helix-forming residues initiating this conformation via direct membrane interaction, resulting in an N-terminal extension of the α-helix. Previous work on the AB1 peptide, comprising the amino acids encoded by Ambn exons 3 and 4 including the 16 N-terminal residues of xAB2N, showed that it does not form a helix in the presence of LUVs of the same composition and size as those used in this study [25], signifying that residues 3 to 20 of AB2 are necessary to initiate α-helix formation.

While residue 24 of AB2 showed a loss of mobility in the presence of LUVs (Figure 1H), a continuation of the helix beyond the two proline residues at positions 20 and 21 would be rather remarkable, as proline residues generally disrupt α-helical structure [51]. However, a non-α-helical contact with LUVs by residue 24 and surrounding residues of AB2 could alternatively be present here and cause some of the immobilization observed in EPR at this site. Moreover, aside from commonly interrupting α-helices [51], proline residues have also been observed to support α-helical structure in hydrophobic environments [52], leading to the likelihood that the membrane-bound state of the AB2 α-helix enabled the perpetuation of the helix past prolines 20 and 21.

Examination of residue immobilization through the labeled peptides AB2 and xAB2N showed that self-assembly and co-assembly with Amel cause Ambn peptides to gain structural order at all sites tested. In addition to direct Ambn–Amel and Ambn–Ambn interactions, Ambn peptides can undergo a change induced by Amel or Ambn that causes the peptides to fold in on themselves, resulting in intramolecular contacts for one or more spin-labeled sites. Furthermore, the observed immobilization in response to Amel or Ambn was most robust in residues within and N-terminal to the self-assembly motif of both peptides (Figure 3A–D and Figure 4A–D). This corresponds with global participation of the N-terminal region of Ambn in Ambn–Amel and Ambn–Ambn interactions, while its Y/F-x-x-Y/L/F-x-Y/F motif is the most immobilized due to its direct mediation of protein–protein assembly. Also corresponding with this model is our confirmation that only the region immediately surrounding the Ambn self-assembly motif showed interactions with Amel (Figure 8A–D), as evidenced by the lack of a fluorescence shift in AB2-W14Y (Figure 8C). This result was in agreement with previous findings using co-immunoprecipitation [28].

While EPR revealed changes in mobility in AB2 and xAB2N in the presence of full-length Ambn, it revealed only minor spectral changes in the presence of AB2 peptide (Figure S3), and AB2 and AB2N similarly did not show statistically significant tryptophan fluorescence peak shifts in the presence of AB3 (Figure 6). These results showing only weak interactions between the peptides do not necessarily signify that peptide–peptide interactions did not take place. Ambn–Ambn interactions at the domain present in AB2 peptide have been shown to occur [27,46], making the occurrence of AB2–AB2 and similar peptide–peptide interactions plausible. For the EPR results, it should be emphasized that very small complexes, such as heterodimers of the peptides, tumble rapidly. Such fast rotational diffusion sharpens the EPR lines of the bound state and could make it difficult to distinguish between the bound and unbound states.

The non-significant results from tryptophan fluorescence measurements of AB2–AB3 and AB2N–AB3 combinations could be due to these peptide–peptide interactions not inducing a significantly detectable change in the environment of AB2 tryptophan residues, as opposed to AB2–LUV and AB2N–LUV interactions (Figure 6A,C,E and elsewhere [25]). It is also possible that the quenching of tryptophan in AB2 by residues such as lysine, tyrosine, glutamine, asparagine, glutamic acid, and histidine [53,54] in AB3 could have flattened the fluorescence peak, leading to a less pronounced redshift than the highly pronounced blueshifts in the presence of LUVs. Alternatively, Ambn–Ambn interaction at the self-assembly region could in fact depend upon the presence of Ambn segments other than those encoded by Ambn exon 5. Those surrounding residues might impart proper orientation of the interacting motif within exon 5 to optimize Ambn self-assembly. This is evidenced by the changes in AB2 and xAB2N structure upon addition of full-length Ambn, as detected using EPR (Figure 4).

Previously, Ambn was found to exist in a range of monomeric and oligomeric forms [55], with its oligomer formation being dependent on its self-assembly domain encoded by exon 5 [46]. Ambn was also found to localize to ameloblast-like cell membranes via a putative helix-forming domain encoded within exon 5 [7]. Therefore, it follows that self-interaction and membrane binding for ameloblast adhesion co-exist in vivo and are both mediated by residues within the same region of Ambn. The finding herein that AB2–membrane interaction is independent of AB2–AB2 interactions or oligomer formation (Figure 7) does not necessarily imply that AB2 peptide or Ambn is in monomeric form when membrane-bound but that its membrane interactions rely upon a mechanism independent of its self-interaction. This mechanism is likely that of forming α-helical insertions into membrane bilayers between phospholipid headgroups. Such a mechanism was previously observed in other membrane-interacting proteins such as BAR domain-containing proteins that also insert α-helical wedges [56,57], proteins that form amyloid upon dysfunction but interact with membranes during their normal functions [49,58,59], and antimicrobial peptides [60]. Taken together with the discovery of the AH formed by the Ambn exon 5-encoded region in the presence of membranes [7,25], our data suggest that the ability to form the observed α-helix is the element that allows AB2 to bind membranes. This amphipathic α-helix formation therefore allows Ambn to adhere ameloblasts to the enamel matrix via direct interaction between Ambn and the ameloblast membrane. Indirect interaction with ameloblast membranes is an alternative possibility, as previous studies have located heparin-, integrin-, and fibronectin-binding domains in Ambn [61,62,63]. However, unlike the directly membrane-interacting N-terminal residues of the region encoded by exon 5, those domains are poorly conserved throughout animal and mammalian evolution [25] and are therefore unlikely to be essential for Ambn function.

Combining Ambn peptide, Amel, and liposomes in one system and performing tryptophan fluorescence and EPR measurements revealed that liposomes and Amel compete for interaction with Ambn. It must be noted that Amel–membrane interactions may also come into play, as CD and fluorescence spectroscopy have previously shown Amel to bind liposomes [64,65]. To measure and compare the binding strengths between Ambn and itself, Ambn and Amel, and Ambn and membranes directly while accounting for the roles of Amel–Amel and Amel–membrane interactions, a more energetically quantitative technique will be needed and is worth undertaking in a future study.

A growing body of evidence supports the proposed role of Ambn as a cell adhesion protein and the importance of the region encoded by exon 5 to this role [3,26]. Deletion of Ambn exons 5 and 6 in a mutant mouse model led to the early detachment of ameloblasts from developing tooth surfaces, leading to defective enamel formation [3,66], underscoring the likelihood of the conserved region in exon 5 adhering these cells to their matrix. Members of a family afflicted with hypoplastic amelogenesis imperfecta were found to carry a deletion of Ambn exon 6 [67], illuminating the importance of this region adjacent to the one encoded by exon 5. Adding exogenous Ambn to ameloblast-like cells in 3D culture led to an increase in their aspect ratio as well as cell clustering, effects that were abrogated upon deletion of the exon 5-encoded region [68]. Deletion of exon 6 also had a negative effect on the Ambn-mediated increase in aspect ratio of those cells, albeit not as drastic an effect as the deletion of exon 5 [68]. Adding exogenous Amel and Ambn together to ameloblast-like cells led to a smaller increase in aspect ratio than did adding Ambn without Amel [68]. Thus, Amel and Ambn did not work synergistically to cause ameloblast polarization, nor did Amel cooperate with Ambn in adhering to the cell membrane. Consistent with our recent observation in 3D cell cultures, the current in vitro study did not show any enhancement of interactions between Ambn peptides and membrane-mimicking LUVs in the presence of Amel (Figure 8 and Figure 9).

Amel–Ambn interactions appear to be critical for proper enamel formation, as mutant mice lacking both Amel and Ambn had significantly more hypoplastic enamel than mice lacking only one of the proteins [69]. Furthermore, Amel and Ambn affect each other’s expression [70], are co-secreted by a common secretory pathway early on during amelogenesis [70] and co-localize in Tomes’s process and in forming enamel [71]. N-terminal fragments of Amel and Ambn also co-localize around enamel rods in maturation stage [6,24]. In vitro experiments using recombinant proteins and synthesized peptides showed that the N-terminal region of Amel interacts directly with the region of Ambn encoded by exon 5 [28,30]. The current findings that show Ambn to interact with membranes and with Amel at the same region pose the question of whether (i) interactions occur on the same Ambn molecule at the same time in vivo or (ii) separate portions of Ambn are allocated to interact with each target. Alternative splicing could be one mechanism facilitating scenario (ii). Humans, pigs, mice, and rats express two isoforms of Ambn, one containing and the other lacking fifteen evolutionarily conserved amino acids at the N-terminal end of the sequence encoded by exon 6 [55,72,73]. It is possible that one isoform preferentially interacts with one target over the other. Another possibility is that controlled Ambn secretion by ameloblasts via secretory vesicles [70] could direct it to interact with one target over the other. By this mechanism, Ambn secreted alone might be driven toward membrane interaction for cell adhesion and Ambn co-secreted with Amel is destined to its enamel-building role implemented via co-assembly with Amel [28].

Based upon the dynamics of Ambn–Amel and Ambn–membrane interactions observed in the current study and the distribution of structural gains detected in xAB2N and AB2 via EPR herein, we propose a mechanism whereby a single molecule of Ambn interacts with the cell membrane and self-assembles or co-assembles with another Ambn or Amel molecule, as in scenario (i). These interactions occur via an Ambn multitargeting domain stretching from mouse ameloblastin residue 57, equivalent to residue 7 of xAB2N, to residue 90, equivalent to residue 24 of AB2 peptide (Table S1). This domain includes the Y/F-x-x-Y/L/F-x-Y/F assembly motif, which is present in Amel and Ambn [27] and situated from residues 1 to 6 of AB2 and 17 to 22 of xAB2N. It also includes the membrane-binding AH motif, which evolutionary analysis has predicted to lie from residues 69 to 86 of mouse Ambn [25]. Interactions with LUVs in the current study appeared to occur roughly equally from residue 7 of xAB2N to residue 24 of AB2 under EPR (Figure 1), suggesting that either the membrane-binding AH motif extends further on both N- and C-terminal sides of the predicted sequence or that Ambn residues 69 to 86 form an AH while surrounding residues in the multitargeting domain form intermediate structures due to proximity to the membrane.

**Scheme 1:**
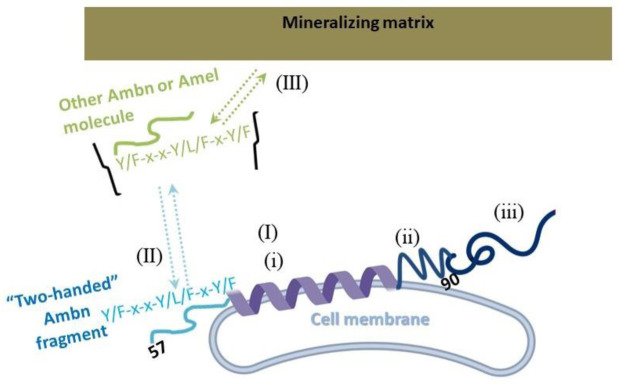
Schematic of proposed mechanism enabling the multitargeting domain of a single Ambn molecule to interact (I) with the plasma membrane of an ameloblast and (II) with Amel or Ambn in the enamel-mineralizing matrix simultaneously. This would be equivalent to amino acids 57 to 90 of mouse ameloblastin (residue numbers shown). In (I), the helix-forming region (i) and the proline-containing domain (ii) bind the membrane while the disordered C-terminal region (iii) coils away. In (II), the Y/F-x-x-Y/L/F-x-Y/F motif remains in proximity to the membrane while interacting with an Amel or other Ambn molecule situated in the matrix (III), thus two-handedly tying the cell membrane to the matrix. Created with BioRender.com (accessed on 20 September 2021).

In contrast to EPR spectra measured in the presence of LUVs, EPR spectra upon addition of Amel or Ambn showed the most robust gains in structure, as well as tertiary/quaternary contacts in the case of Amel, in the self-assembly region of AB2 and xAB2N and the residues N-terminal to the region (Figure 3 and Figure 4). This difference in localization could be due to Ambn–Ambn and Ambn–Amel interactions as well as accompanying structural changes, occurring mainly within and N-terminal to the assembly motif, with membrane interactions initiated by the putative amphipathic α-helix-forming region immediately C-terminal to it. This would enable such a short sequence as amino acids 57 to 90 of mouse Ambn to interact dynamically with membrane and protein partners (Scheme 1). We propose that the helix-forming region established by evolutionary analysis [25] to comprise residues 3 through 20 of AB2 interacts with ameloblast membranes (I), while the self-assembly/co-assembly region, as defined by the Y/F-x-x-Y/L/F-x-Y/F motif, interacts with like motifs on Ambn or Amel (II), resulting in a dynamic balance between the membrane-bound structure of Ambn and additional folding of Ambn on itself or in complex with Amel or other Ambn molecules. Amel and a portion of Ambn interacting with membrane-bound Ambn in turn interact with the mineralizing matrix (III) as they guide enamel formation [23], or the membrane-bound Ambn could also interact with the mineralizing matrix at its C-terminal end. The “two-handedness” or multitargeting ability of membrane-bound Ambn, which allows it to bind cell membranes as well as enamel-forming proteins and the enamel matrix itself, thereby enables adhesion of the membrane to the enamel matrix. In this way, Ambn–membrane interaction does not exclude Ambn–Ambn or Ambn–Amel interaction for each Ambn molecule. Competition between Ambn–membrane interactions and Ambn–Amel interactions could function as a modulator of the cell adhesion and polarization functions of Ambn and its enamel-forming function, where more membrane interactions would upregulate the former and more interactions with Amel would upregulate the latter.

The AB2 and xAB2N peptides, while not natural products of Ambn cleavage during enamel formation [74], have proven highly useful in our recent studies as a model pinpointing the residues encoded within exon 5 that form structural motifs that mediate membrane interactions and Amel–Ambn co-assembly [7,25,28]. The “two-handedness” in Ambn enabled by these residues could also permit it to interact with the cell membrane and with polarization proteins simultaneously. Addition of exogenous Ambn to ameloblast-like cells also regulated the expression of the cell polarization genes Vangl2, Vangl1, Prickle1, ROCK1, and ROCK2 [26]. Furthermore, ameloblast-like cells treated with exogenous Ambn showed asymmetric distribution of the polarization markers E-cadherin, Par3 and Claudin-1 that was dependent upon the presence of the region encoded by Ambn exon 5 [68]. Thus, Ambn supportably promotes a signaling cascade leading to ameloblast polarization, and in so doing it could play a critical role in the formation of Tomes’s processes [26], which are finger-like extensions of secretory ameloblasts’ cytoplasm at their apical end [75]. In view of these proposed adhesion and signaling functions, Ambn could be a matricellular protein, which by definition is a protein that interacts with several functional partners including matrix proteins and membrane proteins in order to modulate cell functions and cell-matrix interactions [76]. Many of these interactions appear to be mediated by isolated motifs localized in the region encoded by Ambn exon 5 as well as the larger multitargeting region of Ambn.

## 4. Materials and Methods

### 4.1. Protein Expression and Purification and Peptide Synthesis

To prepare recombinant porcine Amel with no tryptophan residues (rP172-W25Y/W45Y/W161Y, to be referred to in the text as rP172-W0), the W25Y mutation was generated using a Q5 Site-Directed Mutagenesis Kit (New England Biolabs, Inc., Ipswich, MA, USA) against a previously obtained rP172-W45Y/W161Y background [77]. Subsequently, rP172-W0 was expressed and purified as previously described for recombinant Amel [24,78]. Recombinant mouse Ambn was expressed and purified as previously described [25,79]. Briefly, Ambn was expressed in *E. coli*; purified via nickel affinity columns; dialyzed; enzymatically cleaved in order to release its thioredoxin, histidine, and S-tags; and further purified via high-performance liquid chromatography (HPLC) over an increasing acetonitrile gradient. Purified proteins were lyophilized and kept at −20 °C. BSA was purchased from Sigma (A-4503; St. Louis, MO, USA) and stored at 4 °C prior to use, after which it was dissolved in water at a stock concentration of 10 mg/mL.

The following were synthesized by Biomer Technologies (Pleasanton, CA, USA): Peptides AB2, composed of the 37 residues encoded by exon 5 of Ambn; AB2 variants lacking one or both tryptophan residues; AB2N, comprising the first 20 amino acids of the AB2 sequence; and extended AB2 N-terminus (xAB2N), a 36-amino-acid peptide composed of the last 16 residues before those encoded by exon 5 followed C-terminally by the 20 N-terminal-most residues of AB2. These peptides were rationally designed to evaluate structural changes in residues directly implicated in interactions of Ambn with itself, Amel, and liposomes as well as in residues flanking them. Residues 3–20 in AB2 correspond to residues 69–86 of mouse Ambn and form an AH on membranes [7,25]. Residues 1–6 of AB2 comprise the YSRLGF Ambn self-assembly motif, which was also identified as necessary for its co-assembly with Amel [28]. xAB2N was designed to evaluate structural changes in residues N-terminal to the self-assembly and AH motifs. AB2 and xAB2N cysteine mutants were synthesized and spin-labeled by Biomer Technologies. Paramagnetic spin label (1-oxyl-2,2,5,5-tetramethyl-∆3-pyrroline-3-methyl) methanethiosulfonate (MTSL), catalogue number O875000, was purchased from Toronto Research Chemicals (North York, ON, Canada). All unlabeled and labeled peptides and their variants used in this study are listed in Table S1.

Protein and peptide concentrations were determined by spectrophotometric absorbance at 280 nm. Lyophilized proteins and peptides were dissolved in water and, unless otherwise indicated, were subsequently agitated at 4 °C for 18–42 h before dilution in buffer to the concentration used in each experiment.

### 4.2. LUV Preparation

Chloroform solutions purchased from Avanti Polar Lipids, Inc. (Alabaster, AL, USA), of 1-palmitoyl-2-oleoyl-glycero-3-phosphocholine (POPC), 1-palmitoyl-2-oleoyl-*sn*-glycero-3-phosphoethanolamine (POPE), 1-palmitoyl-2-oleoyl-*sn*-glycero-3-phospho-l-serine (POPS), soy-derived phosphatidylinositol (PI), and sphingomyelin (SM) were mixed at a 40:25:15:10:10 lipid molar ratio to obtain a previously used membrane composition [25] that mimics the epithelial cell membrane [31]. Liposomes, or LUVs, were then prepared from this composition as previously described [25]. Briefly, the chloroform solvent was evaporated, the resulting lipid mixture vacuum was desiccated overnight, and the vesicles were rehydrated in appropriate buffer. The resulting suspension consisted of MLVs, which were utilized in the clearance assay. LUVs were prepared from this MLV suspension via repeated freeze–thaw cycles followed by extrusion through 400 nm diameter and/or 100 nm diameter polycarbonate filters [49]. Liposome size was verified via dynamic light scattering. All experiments incorporating LUVs involved the 100 nm diameter size, except for the leakage assay.

To prepare leakage vesicles, LUVs with a diameter of 400 nm were prepared with a fluorophore, and its quencher was encapsulated within [80]. Briefly, lipids were first rehydrated in 10 mM HEPES, pH 7.4, 2.4 mM KCl, 1 mM EDTA, 3 mM sodium azide, 9 mM 8-aminonaphthalene-1,3,6-trisulfonic acid (ANTS), and 25 mM *p*-xylene-bis(pyridinium bromide) (DPX). This resuspension was subjected to freeze–thaw cycles and extrusion through 400 nm diameter filters. Unencapsulated ANTS and DPX were removed via gel filtration using a HiTrap desalting column (GE Healthcare, Chicago, IL, USA), and LUVs were eluted in a buffer composed of 10 mM HEPES, pH 7.4, 50 mM KCl, 1 mM EDTA, and 3 mM sodium azide.

### 4.3. CD

CD spectra were measured using a Jasco J-815 spectropolarimeter (Jasco Inc., Easton, MD, USA). Samples were placed in a 1 mm quartz cell and were measured every 0.5 nm at a 100-nm/min scan rate, a bandwidth of 1.0 nm, and a digital integration time of 2 s. Eight spectral scans were averaged, the appropriate background was subtracted, and the values were normalized to obtain the mean residue ellipticity (MRE). Each sample was prepared by diluting peptide into 10 mM Tris-HCl, pH 7.5, 50 mM NaCl buffer to a final pH of 7.4 and measuring the CD spectrum. LUVs were subsequently added, and the CD spectrum measured again. Final spectra were smoothed using the means movement method with a convolution width of 11. Spectra resulting from peptide with and without LUVs were compared, and a shift from the negative peak at approximately 195 nm that is indicative of an unstructured polypeptide chain to two negative peaks at 208 and 222 nm [81] characteristic of α-helical structure confirmed peptide–membrane interaction and the resulting formation of an α-helix in the peptide.

### 4.4. EPR

EPR of spin-labeled peptides was measured using a continuous-wave X-band Bruker EMX spectrometer (Bruker Biospin Corp., Billerica, MA, USA) equipped with an ER 4131VT temperature controller and HS cavity. For spectral measurements, samples of 15 μM spin-labeled peptide were loaded into borosilicate glass capillaries (0.6-mm inner diameter × 0.84-mm outer diameter, VitroCom, Mt. Lakes, NJ, USA), and their spectra were recorded at 12.60 milliwatts incident microwave power and 100 G. scan width. Ten to fifteen scans were accumulated for each spectral measurement. Inverse central linewidths (LWs) were measured by marking the distance between the maximum and minimum of the central line in each spectrum in the Bruker EMX WinEPR Acquisition program and then calculating the reciprocal (1/LW). Inverse full widths at half maxima (FWHMs) were calculated, via the Bruker EMX WinEPR Acquisition program, by first integrating EPR spectra directly yielded from experiments to generate their respective absorbance spectra. The center peak was measured from top to baseline for each spectrum and divided by two, whereupon the distance between the sides of the peak was measured at this half-height, and the reciprocal was subsequently calculated (1/FWHM).

For experiments in which a spin-labeled peptide was combined with LUVs, concentrated peptide dissolved in water was diluted to 15 µM in 10 mM Tris, pH 7.4, 50 mM NaCl, to which LUVs suspended in the same buffer were added at the peptide-to-phospholipid molar ratios indicated in Figure 1. For experiments combining a labeled peptide with an unlabeled protein or peptide, concentrated labeled peptide dissolved in water was diluted to 15 µM in 50 mM Tris, pH 7.6, 50 mM NaCl in order to optimize the pH for possible self-assembly or co-assembly. For experiments combining labeled peptide with recombinant Amel, concentrated rP172-W0 previously dissolved in water was added to make 15 µM rP172-W0 for one measurement, followed by another addition of rP172-W0 to a final concentration of 75 µM for the last measurement. For experiments combining labeled peptide and BSA, concentrated BSA dissolved in water was titrated into 15 µM peptide to 0.3 mg/mL followed by 1.5 mg/mL final BSA concentrations, the mass equivalents of 15 and 75 µM Amel. For experiments combining labeled peptide and recombinant Ambn, concentrated Ambn stock solution in water was added to 15 µM peptide to a final concentration of 0.3 mg/mL Ambn. This was the only concentration used due to the difficulty of dissolving Ambn at sufficiently high stock concentrations to yield a 1.5 mg/mL final concentration. For experiments combining labeled peptide with unlabeled AB2, concentrated AB2 in water was titrated into 15 µM labeled peptide to 15 µM followed by 75 µM final unlabeled AB2 concentrations. Experiments using a combination of spin-labeled peptide, LUVs, and rP172-W0 utilized 10 mM Tris, pH 7.4, 50 mM NaCl. Concentrated peptide or protein stock dissolved in water and LUVs suspended in buffer were added in the order indicated, at final concentrations of 15 µM for labeled peptide, 15 µM for rP172-W0, and 600 µM phospholipid concentration for LUVs.

### 4.5. Intrinsic Tryptophan Fluorescence

The tryptophan fluorescence of AB2, AB2-W14Y, AB2N, and xAB2N peptides was measured in the presence or absence of LUVs, of AB3, and/or of rP172-W0 using a Horiba (Kyoto, Japan) Jobin Yvon FluoroLog-3 modular spectrofluorometer or a Hitachi (Tokyo, Japan) F-2500 spectrofluorometer. Final spectra were the averages of three fluorescence emission scans measured from 300 to 400 nm with an excitation wavelength of 295 nm, an excitation slit width of 2.5 or 5 nm, and an emission slit width of 5 nm. Both spectrofluorometers were equipped with a 1 cm path length cuvette, and the appropriate blank spectra were subtracted. The protein or peptide was diluted into 10 mM Tris-HCl, pH 7.5, 50 mM NaCl to a final pH of 7.4. The data, which were representative of at least three independent experiments, were analyzed using Microsoft Excel. *P*-values were determined using one-tailed Student’s *t*-tests and are described in figure legends. The differences were considered significant if *p* < 0.05.

### 4.6. Ambn–Membrane Interactions by Leakage Assay

AB2 was added at varying concentrations to 150 μM 400 nm diameter LUVs in which ANTS and DPX were encapsulated (see above). The release of ANTS and its quencher DPX was measured in a Hitachi F-2500 fluorescence spectrometer every 2 s as an increase in ANTS fluorescence intensity. Excitation and emission wavelengths were set at 380 and 520 nm with slits of 2.5 and 20 nm, respectively, and a response time of 0.08 s. One hundred percent leakage was attained using a final concentration of 0.04% Triton X-100 (EM Science, Gibbstown, NJ, USA), and all data were normalized to fluorescence intensity at this amount. To determine concentration dependence, data were scaled by subtracting the lowest normalized leakage value across all samples from the normalized leakage value of each sample and dividing the resulting value by the difference between the highest and lowest normalized leakage values across all samples; values measured after 15 min of LUV leakage were used.

### 4.7. Ambn–Membrane Interactions by Clearance Assay

Changes in light scattering by MLVs can be detected by measuring their optical density at 500 nm. Light scattering is lost at this wavelength when vesicles become smaller, a clearance that can occur when membrane-interacting proteins are added to MLVs [59]. Thus, clearance of MLVs derived from the suspension of phospholipids used to make LUVs (see above) was monitored by measuring changes in light scattering every 20 s for 30 min at a wavelength of 500 nm in a Beckman Instruments DU 600 Series spectrophotometer (Beckman Coulter, Brea, CA, USA). AB2 at varying concentrations was added to a quartz cuvette containing 150 μM MLVs in 10 mM Tris, pH 7.4, 50 mM NaCl. Water was added to MLVs as a negative control. Data were normalized by setting initial values to 1. In this way, normalized A_500_ at each time point reflected the fraction of remaining light scattering. To determine concentration dependence, data were scaled by subtracting the lowest normalized clearance value (defined as the difference between 1 and the percentage of maximal absorbance value at 500 nm) across all samples from the normalized clearance value of each sample and dividing the resulting value by the difference between the highest and lowest normalized clearance values across all samples; clearance values measured after 15 min of MLV clearance were used.

## 5. Conclusions

We performed electron paramagnetic resonance (EPR) with site-directed spin-labeling (SDSL) on Ambn-derived peptides (AB2, xAB2N). Together with other biophysical techniques, our data collectively indicated competition between Ambn–Amel and Ambn–membrane interactions. Specifically, we found an increase in structure throughout both peptides upon interaction with Amel or full-length Ambn as well as an increase in structure throughout the peptides upon interaction with membrane-mimicking liposomes. The increase in structure in the presence of liposomes is an increase in α-helicity, and peptide–membrane interactions were independent from peptide self-association. Ambn peptides interact less with Amel when combined in the presence of liposomes than in the absence of liposomes, as well as less with liposomes when they are added in the presence of Amel. This study reinforces that multitargeting is at the heart of the complex role of Ambn and centers this role in a domain from amino acids 57 to 90 of mouse Ambn. Ambn interactions with different targets may have implications for its cooperative function with Amel in controlling mineralization and its roles in ameloblast polarization, signaling, and adhesion of ameloblast membranes to the enamel matrix.

## Data Availability

Data supporting reported results will be made available upon request.

## Supplementary Materials

The supporting information can be downloaded at: https://www.mdpi.com/article/10.3390/ijms24043484/s1.

## Abbreviations

AB1	Peptide composed of the 40 amino acids encoded by mouse Ambn exons 3 and 4
AB2	Peptide composed of the 37 amino acids encoded by mouse Ambn exon 5
AB2N	AB2 N-terminus
AB3	Tryptophan-free AB2
AH	Amphipathic helix
Ambn	Ameloblastin
Amel	Amelogenin
ANTS	8-Aminonaphthalene-1,3,6-trisulfonic acid
Amtn	Amelotin
BSA	Bovine serum albumin
CD	Circular dichroism
DPX	*P*-xylene-bis(pyridinium bromide)
EMP	Enamel matrix protein
Enam	Enamelin
EPR	Electron paramagnetic resonance
FWHM	Full width at half maximum
G.	Gauss
HPLC	High-performance liquid chromatography
LUV	Large unilamellar vesicle
LW	Linewidth
MLV	Multilamellar vesicle
MRE	Mean residue ellipticity
MTSL	(1-Oxyl-2,2,5,5-tetramethyl-∆3-pyrroline-3-methyl) methanethiosulfonate
PI	Phosphatidylinositol
POPC	1-Palmitoyl-2-oleoyl-glycero-3-phosphocholine
POPE	1-Palmitoyl-2-oleoyl-*sn*-glycero-3-phosphoethanolamine
POPS	1-Palmitoyl-2-oleoyl-*sn*-glycero-3-phospho-l-serine
rP172-W0	Tryptophan-free recombinant porcine amelogenin
SDSL	Site-directed spin-labeling
SM	Sphingomyelin
xAB2N	Extended AB2 N-terminus
