# Supplementary material for: The Dynamic Interactions of a Multitargeting Domain in Ameloblastin Protein with Amelogenin and Membrane
Source: Int J Mol Sci. 2023 Feb 9;24(4):3484. doi: 10.3390/ijms24043484 (PMC9966149; doi:10.3390/ijms24043484)
Supplement: Supplementary file 1 [file ijms-24-03484-s001.zip › ijms-2149775-supplementary.pdf]

Supporting Information

Table S1. Amino acid sequences of Amel protein and Ambn peptides used.

Protein or peptide notation	Sequence
rP172-W0	PLPPHPGHPGYINFSYEVLTPLKYYQNMIRHPYTSYGYEPMGGY<br>LHHQIIPVVSQQTPQSHALQPHHHIPMVPAQQPGIPQQPMMPLP<br>GQHSMTPTQHHQPNLPLPAQQPFQPQPVQPQPHQPLQPQSPM<br>HPIQPLLPQPPLPPMFSMQSLLPDLPLEAYPATDKTKREEVD
AB2	YSRLGFGKALNSLWLHGLLPPHNSFPWIGPREHETQQ
xAB2N	TMRQLGSLQGLNALSQYSRLGFGKALNSLWLHGLLP
xAB2N-7R1	TMRQLGC*LQGLNALSQYSRLGFGKALNSLWLHGLLP
xAB2N-15R1	TMRQLGSLQGLNALC*QYSRLGFGKALNSLWLHGLLP
xAB2N-18R1	TMRQLGSLQGLNALSQYC*RLGFGKALNSLWLHGLLP
AB2-5R1	YSRLC*FGKALNSLWLHGLLPPHNSFPWIGPREHETQQ
AB2-10R1	YSRLGFGKAC*NSLWLHGLLPPHNSFPWIGPREHETQQ
AB2-12R1	YSRLGFGKALNC*LWLHGLLPPHNSFPWIGPREHETQQ
AB2-19R1	YSRLGFGKALNSLWLHGLC*PPHNSFPWIGPREHETQQ
AB2-24R1	YSRLGFGKALNSLWLHGLLPPHNC*FPWIGPREHETQQ
AB2-35R1	YSRLGFGKALNSLWLHGLLPPHNSFPWIGPREHEC*QQ
AB3	YSRLGFGKALNSLYLHGLLPPHNSFPYIGPREHETQQ
AB2-W14Y	YSRLGFGKALNSLYLHGLLPPHNSFPWIGPREHETQQ
AB2N	YSRLGFGKALNSLWLHGLLP

* denotes labeling with paramagnetic (1-oxyl-2,2,5,5-tetramethyl-Δ3-pyrroline-3-methyl)-methanethiosulfonate (MTSL) spin label. Tryptophan residues in AB2, xAB2N, and AB2N used to measure intrinsic fluorescence and tyrosine residues to which tryptophan residues in AB2 and rP172 are mutated are underlined. The notation "xAB2N-7R1," "AB2-5R1," etc. signifies xAB2N or AB2 with the indicated residue mutated to cysteine and labeled with MTSL.

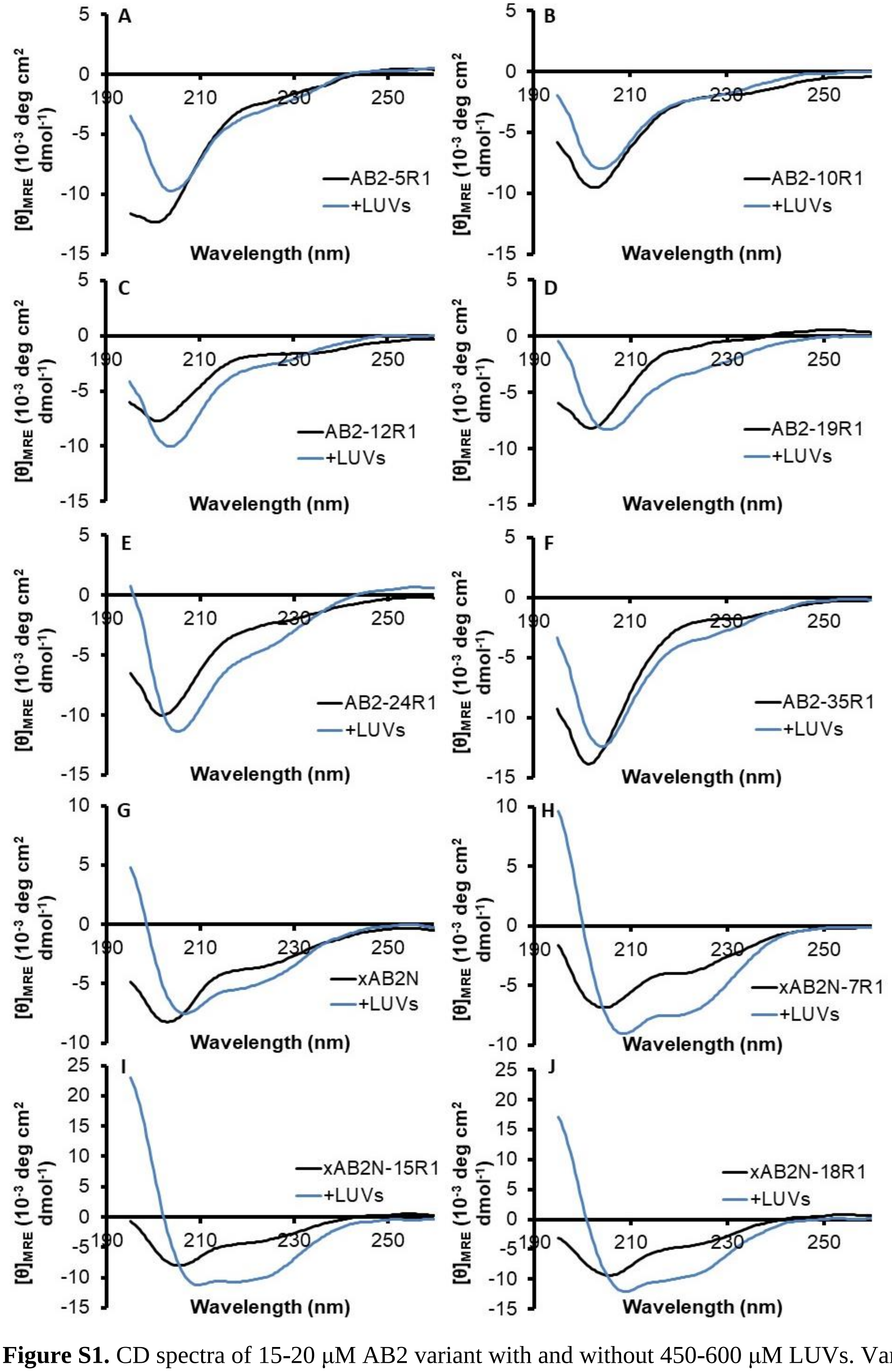

Figure S1. CD spectra of 15-20 μM AB2 variant with and without 450-600 μM LUVs. Variants consist of AB2 with the following residues mutated to cysteine and labeled with MTSL: **(A)** 5, **(B)** 10, **(C)** 12, **(D)** 19, **(E)** 24, and **(F)** 35. CD spectra of 10-15 μM **(G)** unmutated xAB2N and xAB2N with residues

(H) 7, **(I)** 15, and **(J)** 18 mutated to cysteine and labeled with MTSL in the presence or absence of 300-450 µM LUVs.

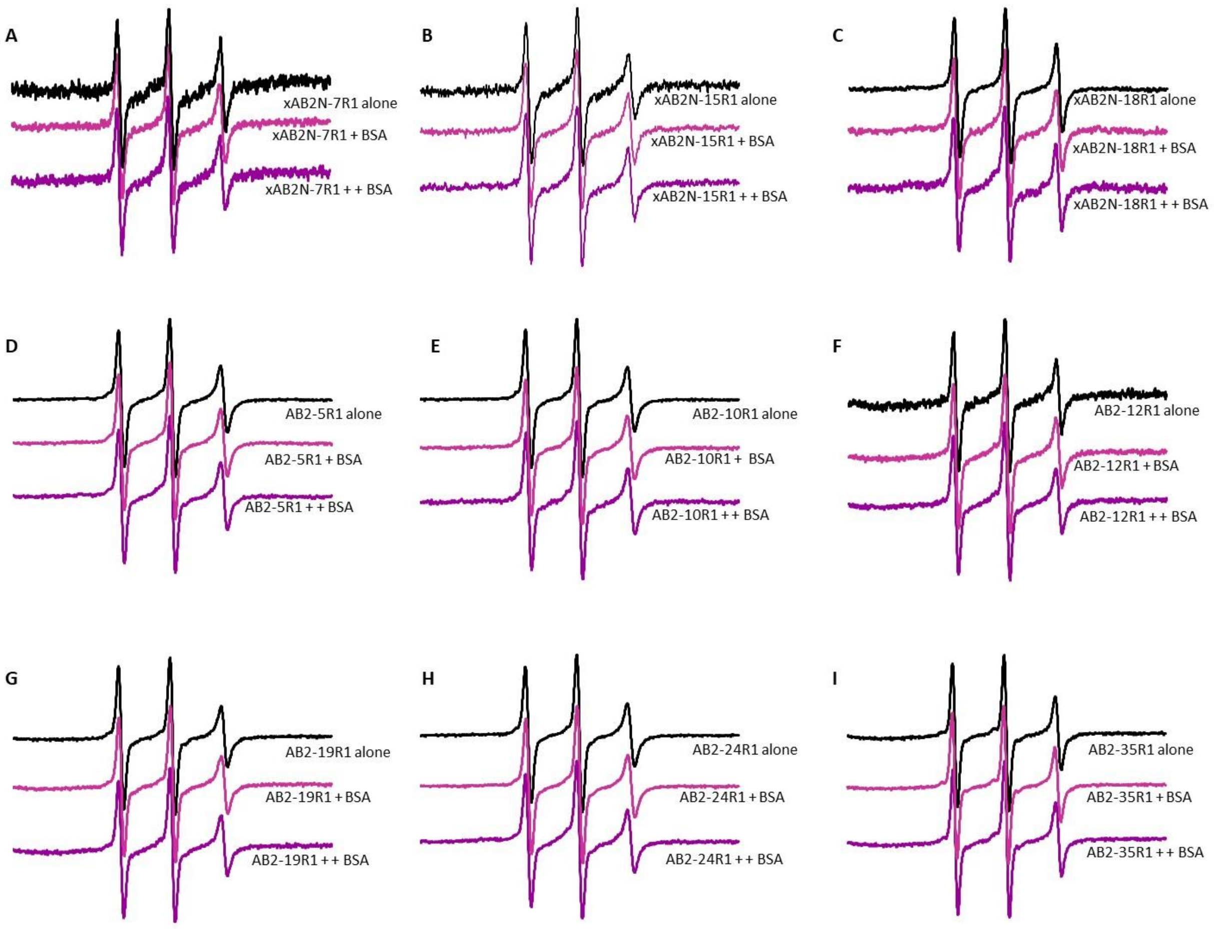

Figure S2. EPR spectra of 15 µM peptide titrated with BSA. Black traces, peptide alone; pink traces, peptide with 0.3 mg/mL BSA added; purple traces, peptide with 1.5 mg/mL BSA added (designated as "+ + BSA"). xAB2N spin-labeled at positions **(A)** 7, **(B)** 15, and **(C)** 18 and AB2 spin-labeled at positions **(D)** 5, **(E)** 10, **(F)** 12, **(G)** 19, **(H)** 24, and **(I)** 35.

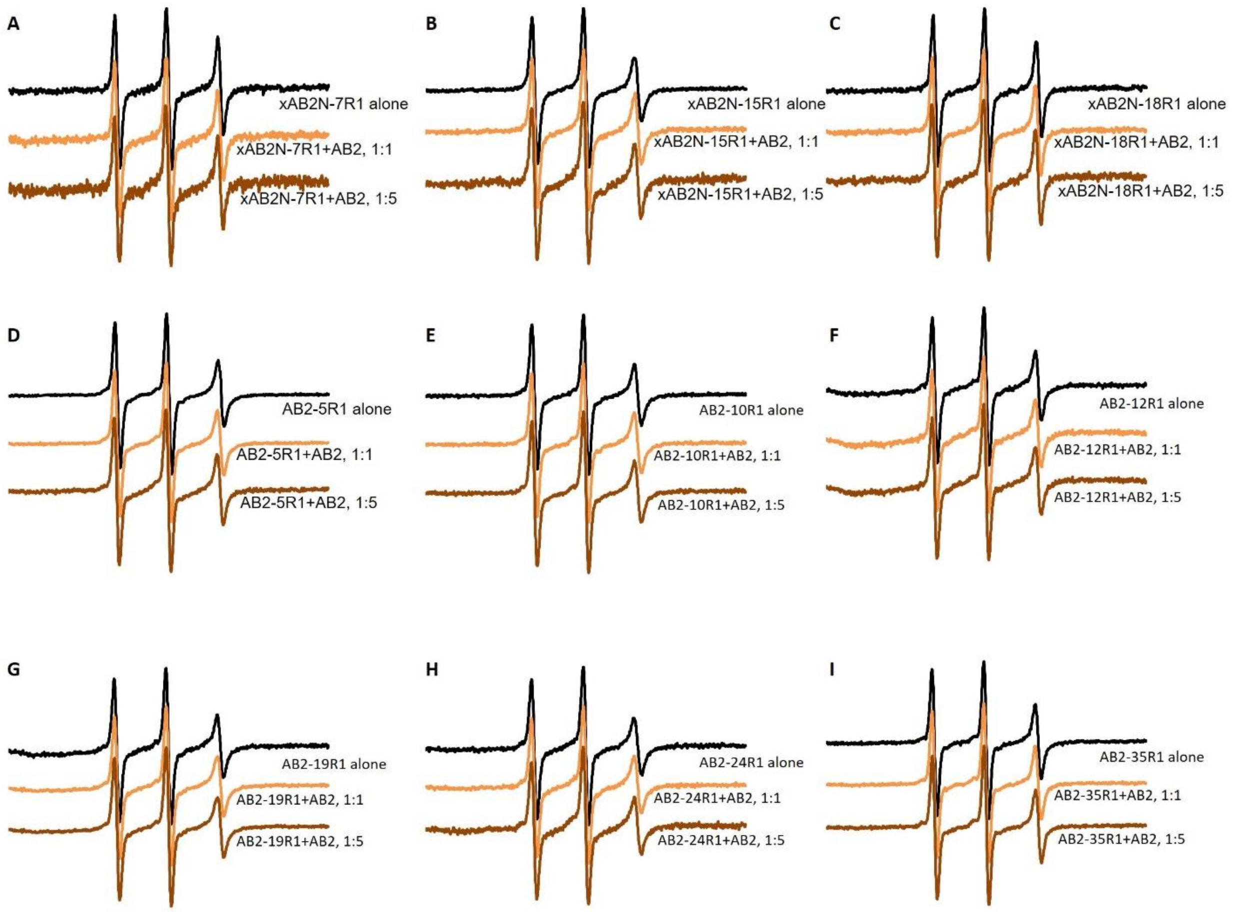

Figure S3. EPR spectra of 15 μM peptide titrated with unlabeled AB2 at indicated labeled peptide-to-unlabeled peptide molar ratios. xAB2N spin-labeled at positions **(A)** 7, **(B)** 15, and **(C)** 18 and AB2 spin-labeled at positions **(D)** 5, **(E)** 10, **(F)** 12, **(G)** 19, **(H)** 24, and **(I)** 35.

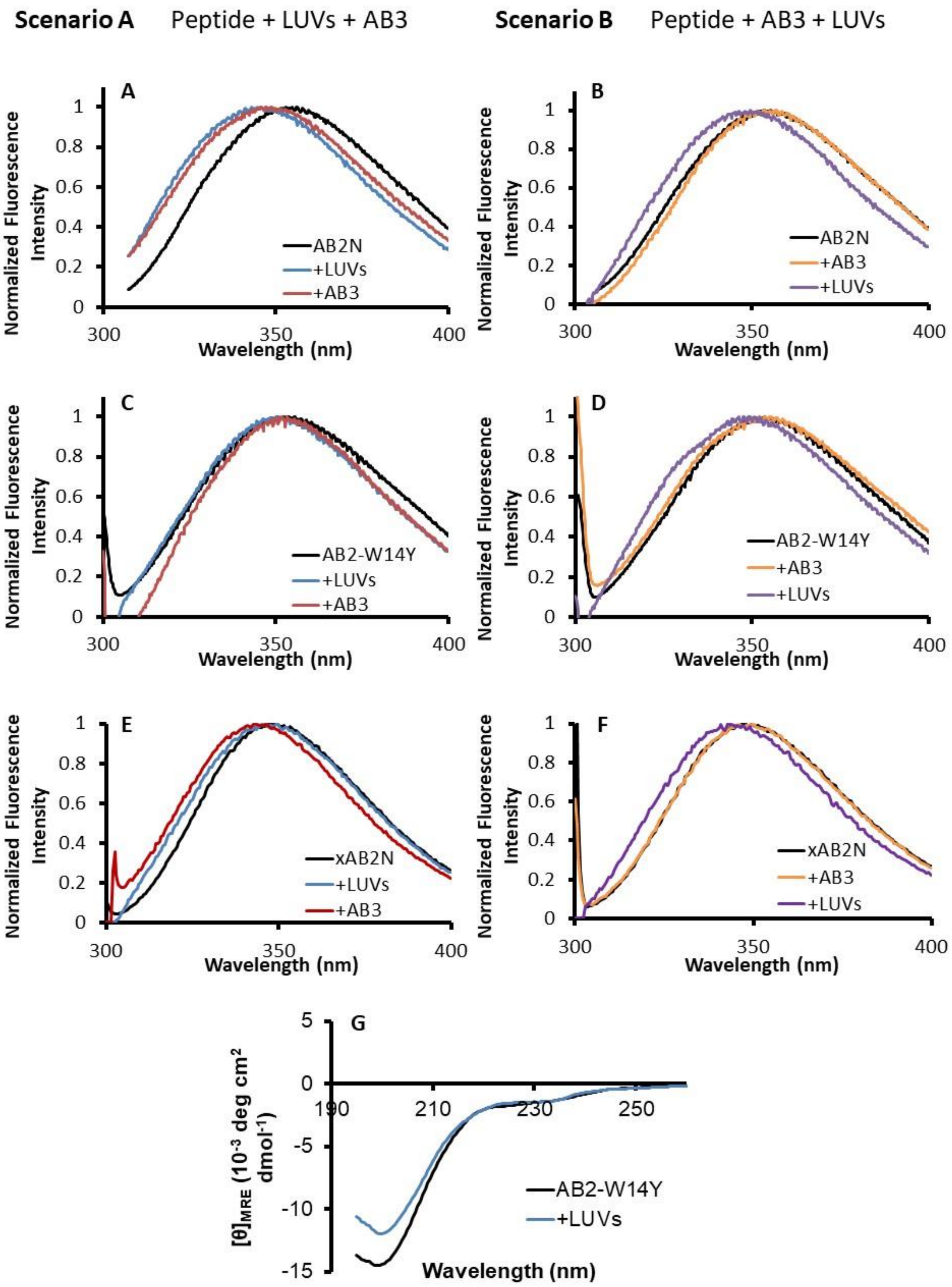

Figure S4. Intrinsic tryptophan fluorescence representative spectra from Scenarios A and B depicted in Figure 5, as follows: **(A)** 8 μM AB2N with 240 μM LUVs, then with 40 μM AB3 subsequently added; **(B**) 8 μM AB2N with 40 μM AB3, then with 240 μM LUVs subsequently added; **(C**) 8 μM AB2-W14Y with 240 μM LUVs, then with 40 μM AB3 subsequently added; **(D)** 8 μM AB2-W14Y with 40 μM AB3, then with 240 μM LUVs subsequently added; **(E)** 8 μM xAB2N with 240 μM LUVs, then with 40 μM AB3 subsequently added; and **(F)** 8 μM xAB2N with 40 μM AB3, then with 240 μM LUVs subsequently added. **(G)** CD spectra of 15 μM AB2-W14Y in the presence or absence of 450 μM LUVs.

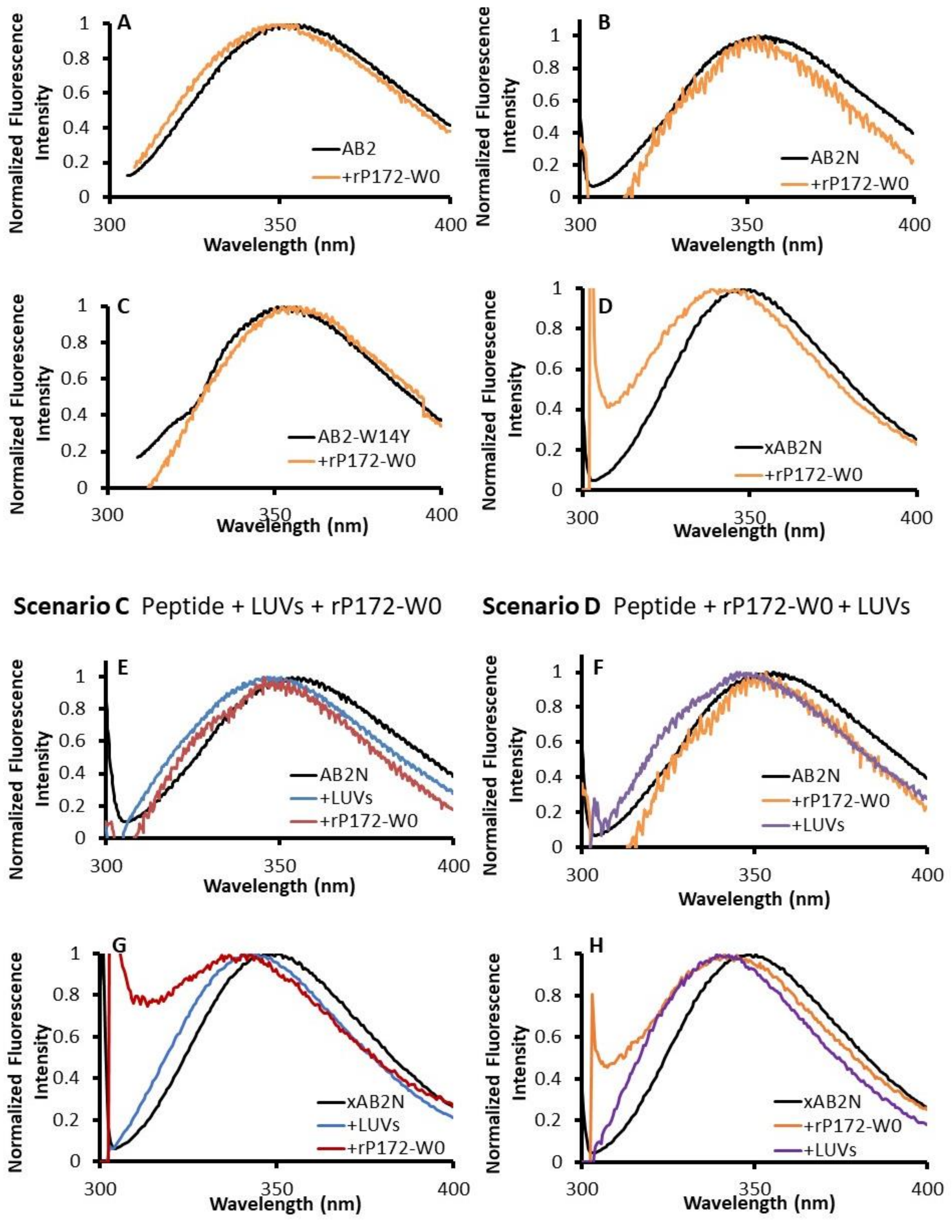

Figure S5. Intrinsic tryptophan fluorescence representative spectra from 8 µM **(A)** AB2, **(B)** AB2N, **(C)** AB2-W14Y, and **(D)** xAB2N before and after addition of 40 µM rP172-W0. Representative spectra for AB2N and xAB2N under Scenarios C and D depicted in Figure 5, as follows: **(E)** 8 µM AB2N with 240 µM LUVs, then with 40 µM rP172-W0 subsequently added; **(F)** 8 µM AB2N with 40 µM rP172-W0, then with 240 µM LUVs subsequently added; **(G)** 8 µM xAB2N with 240 µM LUVs, then with 40 µM rP172-W0 subsequently added; **(H)** 8 µM xAB2N with 40 µM rP172-W0, then with 240 µM LUVs subsequently added.
